# Editorial: Phosphonate chemistry in drug design and development, Volume II

**DOI:** 10.3389/fchem.2022.1054145

**Published:** 2022-11-23

**Authors:** Petri A. Turhanen, Pawel Kafarski

**Affiliations:** ^1^ School of Pharmacy, University of Eastern Finland, Kuopio, Finland; ^2^ Department of Bioorganic Chemistry, Wroclaw University of Science and Technology, Wroclaw, Poland

**Keywords:** organophosphorus, phosphonate, nucleotides, prodrug, bisphosphonate

**FIGURE 1:**
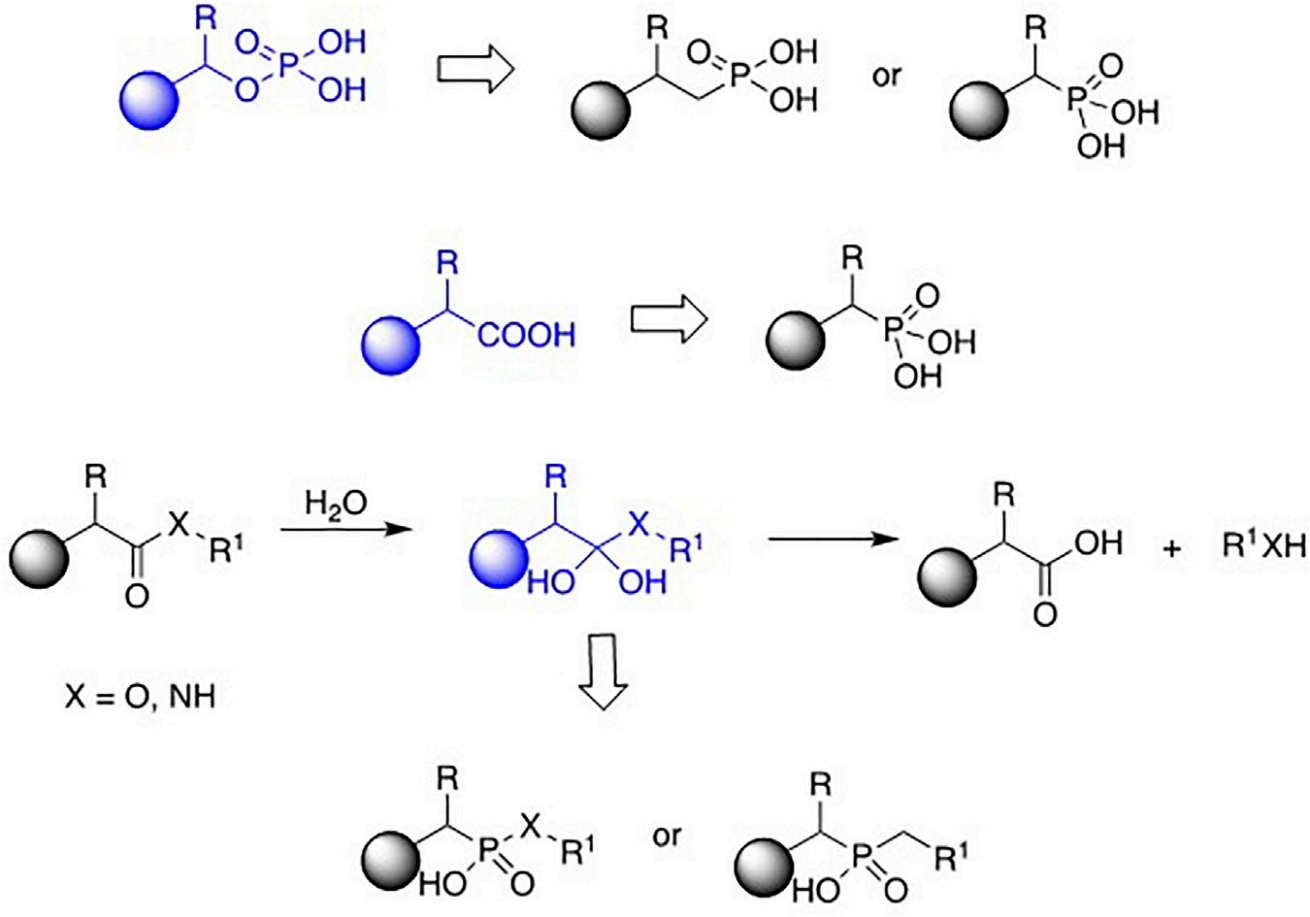
Phosphonic acids as analogues of phosphates, amino acids and peptides.

Phosphonic acid derivatives (including phosphonic, phosphinic, and phosphinous acids) are a diverse group of analogues of natural compounds. The phosphonate moiety replaces the phosphate fragment of organic phosphates, mimics carboxylate moiety in amino- and hydroxy-acids, and acts as a transition state analogue appearing upon hydrolysis of the esters, amides, and peptides (Figure 1). They also quite frequently exhibit interesting and useful physiologic activities and thus are considered potential therapeutics or pesticides. Indeed, some of them have found applications in modern medicine to mention only herbicides and antiviral, antibacterial, and antiosteoporotic drugs.

The current Research Topic is a short supplementation of the preceding one and is composed of two review articles and two original papers. Reviews consider the use of non-hydrolysable phosphate mimics as prodrugs and synthesis and some physiologic activities of amino- and hydroxy-phosphonates and bisphosphonates.

Krečmerová et al. present a comprehensive account of the works performed at the Institute of Organic Chemistry and Biochemistry in Prague on the synthesis and evaluation of non-hydrolysable nucleoside mimics. The Institute is well recognized for synthesis and studies on acyclic nucleoside phosphonates, including popular antiviral agents such as adefovir, tenofovir, and cidofovir, and their pro-drugs. Besides these, special attention is paid to new biologically active molecules with respect to emerging infections and the rising resistance of many pathogens against standard treatments. These new structures include 2,4-diamino-6-[2 (phosphonomethoxy)ethoxy]-pyrimidines, acyclic nucleoside phosphonates with 5-azacytosine as a base moiety, fluorinated in a side chain or aza/deazapurine derivatives. The second class of potential antivirals that are reviewed considers derivatives of non-nucleoside phosphonates such as 2-(phosphonomethyl)pentanedioic acid. Their possible applications, including as anticancer and anti-parasitic agents, were also mentioned.

Kaboudin et al. discuss phosphonates and bisphosphonates, substituted with amine or hydroxyl at *α*-carbon, as stable analogs of phosphates and pyrophosphates. Synthetic procedures leading to these compounds were briefly introduced followed by a detailed description of their possible applications in medicine (anti-proliferative, anti-osteoporotic, and as drug targets to bone tissue), inhibitory activity against variable enzymes, and in bio-imaging.

Two original research articles relate to the selective inhibition of tyrosyl-DNA phosphodiesterase 1 and 2-oxoglutarate and 2-oxoadipate dehydrogenases by rationally designed phosphonates based on the crystal structure of previously described inhibitors.

Tyrosyl-DNA phosphodiesterase 1 repairs stalled type I topoisomerase DNA complexes by hydrolyzing the phosphodiester bond between the topoisomerase and its DNA substrate. The elegant design of novel inhibitors of this process are presented and the crystal structures of the tyrosyl-DNA phosphodiesterase 1 complex with a subset of phosphonate inhibitors are described, progressing research on the binding interactions of these inhibitors with the enzyme.

Succinyl phosphonate and adipoyl phosphonate are established as specific inhibitors of cognate 2-oxo acid dehydrogenases. The present work develops the action of triethyl esters of these compounds towards rats, by studying their membrane-penetrating abilities and influence on the rat brain metabolism (activity against chosen enzymes, influence on the level of certain amino acids), and animal behavior.

These papers alongside the articles previously published in the first volume (https://www.frontiersin.org/research-topics/13124/phosphonate-chemistry-in-drug-design-and-development) of the series have indicated that the interest in synthesis and evaluation of biological activities of phosphonates is a substantial part of organophosphorus medicinal chemistry and that there is still a lot of space for valuable and interesting discoveries.

